# Supplementary material for: Regulation of Gene Expression of phiEco32-like Bacteriophage 7-11
Source: Viruses. 2022 Mar 8;14(3):555. doi: 10.3390/v14030555 (PMC8948821; doi:10.3390/v14030555)
Supplement: Supplementary file 1 [file viruses-14-00555-s001.zip › viruses-1559845-supplementary.pdf]

Supplementary data

Regulation of Gene Expression of phiEco32-like Bacteriophage 7-11

Daria Lavysh [1,*], Vladimir Mekler [2], Evgeny Klimuk [1] and Konstantin Severinov [1,2,3,*]

[1]Institute of Molecular Genetics of National Research Centre «Kurchatov Institute», 123182 Moscow, Russia

[2]Waksman Institute for Microbiology and Department of Molecular Biology and Biochemistry, Rutgers, State University of New Jersey, Piscataway, NJ 08854, USA

[3]Skolkovo Institute of Science and Technology, Center for Life Sciences, 121205 Skolkovo, Russia

Supplementary Table S1. Primers used for transcription and primer extension experiments.

Number "x" in a primer name "Px" indicates primers used to obtain a PCR fragment, containing SaPh711_gp47-promoter x. Primers named "Px_pe" were also used for primer extension reactions. Primers labeled as "s70_" correspond to σ^{70} promoters.

**Primer**	**sequence**
P1	GAT ATC CCA TCT ATC AGT GT
P1_pe	CCT GCT GTA TTA CCC TTG
P8	GCG TAA TTC CTT CAA TTT CC
P8_pe	AAG GTC GTA AGA AAC TAA AGT AG
P16	CAT CCG ACT ACT CCT TAA G
P16_pe	ACA TAG GTA TTG AGA CCC AC
P22	ACC TAG TGA CCA AGA CCA GC
P22_pe	GAA CGG AGT TGA TTC ATG CG
P28	AGG GTG TCA GAG ACC TCA GC
P28_pe	AGT TGC GTT CCC AGA AGT CTT TA
P48	CAT TTG CCG GTG ATT TC
P48_pe	CGC GCA TCA TAT ACA TCG
P69	TAA TGT ATC GTC GAG TCA CAC GC
P69_pe	CGT CAC GCA TTT TAA AAC ACC
s70_P151_1	TAC AAA CCC TTG TAC TAG TG
s70_P151_1_pe	TAA GAA ACA ACG TTT CGA AC
s70_P151_2,3	ATA TTC AAT TCA TCG GGA GG
s70_P151_2,3_pe	ACC TAT TTA CTA ACA TCG CC
s70_P42	GGC TAA AGT TGT CGA TGT TG
s70_P42_pe	GCG GCC AGA AGA ACA T
s70_P99	CCT TGA AAG GTC CGT AGT C

s70_P99_pe	GGT AGC ACG ACC GAT TAC
S.N._PompX_pe	AAC AGA ACG TTT CCA TAT CG
gp47_pe	GTT TTC TTA GGA GTT GGA GCA C

Supplementary Table S2. List of all homologues genes among phiEco32-like phages.

The result of searchers of core genes of the phiEco32 subgroup phages and core genes of the 7-11 subgroup of phages are presented.

7-11 **NC_015938**	GAP52 **NC_019402**	phiEco32 **NC_010324**	NJ01 **NC_018835**	KBNP1711 **KF981730**	ECBP2 **NC_018859**	SU10 **KM044272**	172-1 **KP308307**
SaPh711_gp001	D858_gp001	phi32_6	NJ01_016	ECBP3_0004	ECBP2_0004	SU10_04	1721_23
SaPh711_gp004	D858_gp112	phi32_7	NJ01_017	ECBP3_0005	ECBP2_0005	SU10_05	1721_24
SaPh711_gp005	D858_gp111	phi32_8	NJ01_018	ECBP3_0006	ECBP2_0006	SU10_06	1721_25
SaPh711_gp007	D858_gp108	phi32_10	NJ01_020	ECBP3_0008	ECBP2_0008	SU10_08	1721_27
SaPh711_gp012	D858_gp102	phi32_13	NJ01_023	ECBP3_0011	ECBP2_0011	SU10_11	1721_30
SaPh711_gp021	D858_gp093	phi32_14	NJ01_024	ECBP3_0012	ECBP2_0012	SU10_12	1721_31
SaPh711_gp017	D858_gp097	phi32_18	NJ01_028	ECBP3_0016	ECBP2_0016	SU10_16	1721_35
SaPh711_gp018	D858_gp096	phi32_19	NJ01_029	ECBP3_0017	ECBP2_0017	SU10_17	1721_36
SaPh711_gp020	D858_gp094	phi32_24	NJ01_034	ECBP3_0022	ECBP2_0022	SU10_22	1721_41
SaPh711_gp024	D858_gp090	phi32_26	NJ01_038	ECBP3_0024	ECBP2_0024	SU10_24	1721_43
SaPh711_gp045	D858_gp075	phi32_33	NJ01_043	ECBP3_0029	ECBP2_0028	SU10_29	1721_49
SaPh711_gp047	D858_gp073	phi32_36	NJ01_046	ECBP3_0032	ECBP2_0031	SU10_32	1721_52
SaPh711_gp048	D858_gp072	phi32_40	NJ01_049	ECBP3_0036	ECBP2_0035	SU10_37	1721_55
SaPh711_gp068	D858_gp061	phi32_58	NJ01_067	ECBP3_0056	ECBP2_0054	SU10_56	1721_72
SaPh711_gp079	D858_gp050	phi32_65	NJ01_077	ECBP3_0065	ECBP2_0061	SU10_64	1721_82
SaPh711_gp073	D858_gp052	phi32_67	NJ01_081	ECBP3_0069	ECBP2_0065	SU10_66	1721_86
SaPh711_gp086	D858_gp044	phi32_77	NJ01_092	ECBP3_0080	ECBP2_0075	SU10_78	1721_97
SaPh711_gp099	D858_gp036	phi32_80	NJ01_096	ECBP3_0083	ECBP2_0079	SU10_82	1721_100
SaPh711_gp101	D858_gp034	phi32_82	NJ01_098	ECBP3_0085	ECBP2_0081	SU10_84	1721_102
SaPh711_gp102	D858_gp033	phi32_83	NJ01_099	ECBP3_0086	ECBP2_0082	SU10_85	1721_103
SaPh711_gp103	D858_gp032	phi32_84	NJ01_100	ECBP3_0087	ECBP2_0083	SU10_86	1721_104
SaPh711_gp104	D858_gp031	phi32_85	NJ01_101	ECBP3_0088	ECBP2_0084	SU10_087	1721_105
SaPh711_gp105	D858_gp030	phi32_86	NJ01_102	ECBP3_0089	ECBP2_0085	SU10_088	1721_106

	phi32_4	NJ01_004	ECBP3_0002	ECBP2_0002	SU10_02	1721_21
	phi32_5	NJ01_015	ECBP3_0003	ECBP2_0003	SU10_03	1721_22
	phi32_9	NJ01_019	ECBP3_0007	ECBP2_0007	SU10_07	1721_26
	phi32_12	NJ01_022	ECBP3_0010	ECBP2_0010	SU10_10	1721_29
	phi32_15	NJ01_025	ECBP3_0013	ECBP2_0013	SU10_13	1721_32
	phi32_16	NJ01_026	ECBP3_0014	ECBP2_0014	SU10_14	1721_33
	phi32_21	NJ01_031	ECBP3_0019	ECBP2_0019	SU10_19	1721_38
	phi32_23	NJ01_033	ECBP3_0021	ECBP2_0021	SU10_21	1721_40
	phi32_30	NJ01_041	ECBP3_0027	ECBP2_0026	SU10_27	1721_47
	phi32_32	NJ01_042	ECBP3_0028	ECBP2_0027	SU10_28	1721_48
	phi32_38	NJ01_047	ECBP3_0034	ECBP2_0033	SU10_34	1721_53
	phi32_42	NJ01_050	ECBP3_0037	ECBP2_0036	SU10_39	1721_56
	phi32_44	NJ01_054	ECBP3_0040	ECBP2_0039	SU10_42	1721_60
	phi32_47	NJ01_057	ECBP3_0044	ECBP2_0043	SU10_45	1721_62
	phi32_48	NJ01_058	ECBP3_0045	ECBP2_0044	SU10_46	1721_63
	phi32_49	NJ01_059	ECBP3_0046	ECBP2_0045	SU10_47	1721_64
	phi32_54	NJ01_063	ECBP3_0053	ECBP2_0051	SU10_52	1721_68
	phi32_56	NJ01_065	ECBP3_0054	ECBP2_0052	SU10_53	1721_70
	phi32_57	NJ01_066	ECBP3_0055	ECBP2_0053	SU10_54	1721_71
	phi32_66	NJ01_079	ECBP3_0067	ECBP2_0063	SU10_65	1721_84
	phi32_69	NJ01_083	ECBP3_0071	ECBP2_0067	SU10_68	1721_88
	phi32_70	NJ01_084	ECBP3_0072	ECBP2_0068	SU10_69	1721_89
	phi32_73	NJ01_088	ECBP3_0076	ECBP2_0071	SU10_73	1721_93
	phi32_76	NJ01_091	ECBP3_0079	ECBP2_0074	SU10_77	1721_96
	phi32_78	NJ01_094	ECBP3_0081	ECBP2_0077	SU10_80	1721_98
	phi32_79	NJ01_095	ECBP3_0082	ECBP2_0078	SU10_81	1721_99
	phi32_81	NJ01_097	ECBP3_0084	ECBP2_0080	SU10_83	1721_101
	phi32_87	NJ01_103	ECBP3_0090	ECBP2_0086	SU10_089	1721_107
	phi32_88	NJ01_104	ECBP3_0091	ECBP2_0087	SU10_090	1721_108

		phi32_89	NJ01_105	ECBP3_0092	ECBP2_0088	SU10_091	1721_109
		phi32_90	NJ01_106	ECBP3_0093	ECBP2_0089	SU10_092	1721_110
		phi32_93	NJ01_108	ECBP3_0096	ECBP2_0091	SU10_094	1721_112
		phi32_94	NJ01_109	ECBP3_0097	ECBP2_0093	SU10_095	1721_113
		phi32_95	NJ01_110	ECBP3_0095	ECBP2_0095	SU10_096	1721_114
		phi32_101	NJ01_115	ECBP3_0101	ECBP2_0101	SU10_100	1721_120
		phi32_107	NJ01_123	ECBP3_0107	ECBP2_0104	SU10_105	1721_127
		phi32_108	NJ01_124	ECBP3_0108	ECBP2_0105	SU10_106	1721_128
		phi32_112	NJ01_130	ECBP3_0112	ECBP2_0108	SU10_110	1721_2
		phi32_114	NJ01_133	ECBP3_0114	ECBP2_0109	SU10_112	1721_5
		phi32_115	NJ01_134	ECBP3_0115	ECBP2_0110	SU10_113	1721_6
		phi32_116	NJ01_136	ECBP3_0116	ECBP2_0113	SU10_115	1721_8
		phi32_120	NJ01_001	ECBP3_0119	ECBP2_0115	SU10_117	1721_12
		phi32_123	NJ01_004	ECBP3_0121	ECBP2_0117	SU10_119	1721_15
		phi32_124	NJ01_005	ECBP3_0122	ECBP2_0118	SU10_120	1721_16
		phi32_127	NJ01_009	ECBP3_0125	ECBP2_0119	SU10_124	1721_19
		phi32_128	NJ01_010	ECBP3_0126	ECBP2_0120	SU10_125	1721_20
SaPh711_gp008	D858_gp107	phi32_11					
SaPh711_gp023	D858_gp091	phi32_25					
SaPh711_gp097	D858_gp039	phi32_29					
SaPh711_gp053	D858_gp067	phi32_43					
SaPh711_gp063	D858_gp064	phi32_53					
SaPh711_gp078	D858_gp051	phi32_68					
SaPh711_gp081	D858_gp049	phi32_72					
SaPh711_gp082	D858_gp047	phi32_74					
SaPh711_gp084	D858_gp046	phi32_75					
SaPh711_gp111	D858_gp023	phi32_117					
SaPh711_gp037	-	phi32_35					
SaPh711_gp052	-	phi32_47					

SaPh711_gp080	-	phi32_60
SaPh711_gp076	-	phi32_61
SaPh711_gp074	-	phi32_64
SaPh711_gp113	-	phi32_92
SaPh711_gp116	-	phi32_99
SaPh711_gp135	-	phi32_112
SaPh711_gp003	D858_gp113	-
SaPh711_gp006	D858_gp109	-
SaPh711_gp009	D858_gp106	-
SaPh711_gp010	D858_gp104	-
SaPh711_gp011	D858_gp103	-
SaPh711_gp013	D858_gp101	-
SaPh711_gp014	D858_gp100	-
SaPh711_gp015	D858_gp099	-
SaPh711_gp016	D858_gp098	-
SaPh711_gp019	D858_gp095	-
SaPh711_gp022	D858_gp092	-
SaPh711_gp028	D858_gp086	-
SaPh711_gp029	D858_gp087	-
SaPh711_gp030	D858_gp088	-
SaPh711_gp033	D858_gp082	-
SaPh711_gp040	D858_gp081	-
SaPh711_gp041	D858_gp058	-
SaPh711_gp042	D858_gp078	-
SaPh711_gp043	D858_gp077	-
SaPh711_gp044	D858_gp076	-
SaPh711_gp046	D858_gp074	-
SaPh711_gp049	D858_gp070	-
SaPh711_gp050	D858_gp069	-

SaPh711_gp064	D858_gp063	-
SaPh711_gp065	D858_gp062	-
SaPh711_gp069	D858_gp059	-
SaPh711_gp071	D858_gp055	-
SaPh711_gp072	D858_gp053	-
SaPh711_gp083	D858_gp083	-
SaPh711_gp085	D858_gp045	-
SaPh711_gp094	D858_gp043	-
SaPh711_gp095	D858_gp041	-
SaPh711_gp098	D858_gp037	-
SaPh711_gp100	D858_gp035	-
SaPh711_gp106	D858_gp029	-
SaPh711_gp107	D858_gp028	-
SaPh711_gp108	D858_gp026	-
SaPh711_gp109	D858_gp024	-
SaPh711_gp119	D858_gp016	-
SaPh711_gp120	D858_gp015	-
SaPh711_gp122	D858_gp014	-
SaPh711_gp129	D858_gp013	-
SaPh711_gp130	D858_gp012	-
SaPh711_gp142	D858_gp011	-
SaPh711_gp143	D858_gp010	-
SaPh711_gp144	D858_gp009	-

Supplementary Table S3. Sequences of predicted late and middle promoters in the phiEco32-like subgroup of phages.

Sequences of predicted promoter consensus elements are indicated on the left; late promoters are indicated by red-colored font, middle promoter promoters are indicated in green. Promoters numbers match the number of a gene in front of which the promoter is located.

	phiEco32	NJ01	ECBP2	KBNP1711	SU10	172-1
TAATGTAgA	P_{6}	P_{16}	P_{4}	P_{4}	P_{4}	P_{23}
TAATGTATA	P_{13}	P_{23}	P_{11}	P_{11}	P_{11}	P_{30}
TAATGTATA	P_{26}	P_{38}	P_{24}	P_{24}	P_{24}	P_{43}
TAATGTATA	P_{40}	P_{49}	P_{35}	P_{35}	P_{37}	P_{55}
TAATGTATA	P_{58}	P_{67}	P_{54}	P_{56}	P_{56}	P_{72}
aAATGTATA	P_{68}	P_{82}	P_{66}	P_{70}	P_{68}	P_{87}

Supplementary Table S4. Sequences of predicted late promoters in the 7-11 and GAP52 phge genomes subgroup of phages.

Promoter numbers match the number of a gene, before which the promoter is located. Promoters with lower levels of matching to consensus sequence are highlighted with a grey-colored font.

7-11		GAP52	
P_{1}	GTAATG -(16)- gCTA	P_{1}	GTAATa -(16)- ACTA
P_{8}	GTAAGG -(16)- ACTA	P_{107}	GTAATa -(16)- ACTA
P_{12}	GTAATT -(16)- ACTA	P_{98}	GTAATc -(16)- ACTA
P_{16}	GTAATT -(16)- ACTA	P_{92}	GTAATa -(16)- ACTA
P_{22}	GTAAGG -(16)- ACTA	P_{86}	GTAAGa -(16)- ACTA
P_{28}	GTAAGG -(16)- ACTA	P_{71}	GTAAGa -(16)- ACTA
P_{48}	GTAAGT -(16)- ACTA	P_{59}	GTAAGa -(16)- ACTA
P_{69}	GTAAGT -(16)- ACTA	P_{48}	GTAAGG -(16)- ACTA
P_{84}	GTAATG -(17)- ACTA	P_{46}	GTAATG -(16)- ACTA
